# Nanoarchitectonics for Advancing Bone Graft Technology: Integration of Silver Nanoparticles Against Bacteria and Fungi

**DOI:** 10.3390/microorganisms12122616

**Published:** 2024-12-17

**Authors:** Leticia Ramos Dantas, Maria Alice Witt, Everdan Carneiro, Felipe Francisco Tuon

**Affiliations:** 1 Laboratory of Emerging Infectious Diseases, School of Medicine, Pontifícia Universidade Católica do Paraná, Curitiba 80215-901, PR, Brazil; leticia.dantas@pucpr.edu.br; 2 Chemistry Department, Pontifícia Universidade Católica do Paraná, Curitiba 80215-901, PR, Brazil; maria.witt@pucpr.br; 3 Odontology School, Pontifícia Universidade Católica do Paraná, Curitiba 80215-901, PR, Brazil; everdan.carneiro@pucpr.br

**Keywords:** biofilm, silver, bone, nanotechnology, impregnation, bacteria

## Abstract

Silver nanoparticles have garnered significant attention for their antimicrobial applications. The aim of this study was to develop and characterize a silver nanoparticle-enhanced bone graft and assess its antimicrobial and antibiofilm activities. Bone granules from bovine cancellous femur were impregnated with silver nanoparticles (50 nm). The antimicrobial and antibiofilm activity was tested against various pathogens, including Staphylococcus aureus, Pseudomonas aeruginosa, Candida albicans, Enterococcus faecalis, Acinetobacter baumannii, and Escherichia coli. Biocompatibility and resorption were evaluated in a mouse calvaria model. All the tested pathogens showed susceptibility to silver nanoparticles, with minimal inhibitory concentrations ranging from 0.25 to 4 mg/L. The silver nanoparticle scaffolds demonstrated a significant reduction in biofilm formation across all microorganisms. The graft exhibited a biocompatibility comparable to that of autologous bone, with reduced resorption rates. Additionally, the presence of nanoparticles did not impact radiolucency, and cytotoxicity remained minimal. Bone grafts impregnated with silver nanoparticles effectively reduce biofilm formation, suggesting their potential as a strategic material for various implant applications.

## 1. Introduction

The repair and reconstruction of bone defects are critical challenges in both the medical and dental fields. Bone grafting serves as a pivotal approach to restoring bone loss caused by a variety of conditions, including periodontal disease, trauma, tooth loss, alveolar ridge preservation following tooth extraction, and bone augmentation in the maxillary sinuses [1,2,3]. Bone tissue is the second most frequently transplanted tissue, with the United States and some European countries having the highest demand, accounting for about half a million cases of bone grafting needed annually [4]. In the United States, allografts are the most used, while in Europe, bovine xenografts are more commonly employed [5].

Bovine bone scaffolds are an alternative to autologous grafts that require a second surgical site. A second surgical procedure to obtain bone graft increases morbidity, risk of infection, hemorrhage, and peripheral nerve lesions [6]. Bovine bone grafts have a lower production cost than other grafts, are available on the market, and have good osteoinduction, osteoconduction, and mechanical stability characteristics [5,7].

Several antibiotics and other molecules (e.g., bioglass or metals) have been used to impregnate the implant surface or even the bone graft [8]. While antibiotics can be used for the impregnation of bone grafts, as is commonly practiced in orthopedics, the growing challenge of antimicrobial resistance poses significant limitations. The frequent use of antibiotics such as vancomycin and gentamicin in orthopedic materials may become ineffective in the current landscape of multidrug resistance. In this context, the use of metals emerges as a more attractive alternative, potentially circumventing the induction of resistance associated with antibiotics used in infection treatment [9].

The antimicrobial activity of silver nanoparticles (AgNPs) has been extensively investigated, including incorporating this metal into some dental materials, like tissue conditioners, acrylic resins, and mouthrinses [10]. The small size of the AgNP increases the contact area with the bacterial cell membrane, increasing the permeability and intracellular concentration of silver [11]. Silver ions interfere with the mitochondrial respiratory chain, causing oxidative reduction [12].

In theory, the impregnation of bovine bone scaffolds can decrease trans- and postoperative infection rates, which lead to a high risk of loss of bone volume and chronic infectious processes, such as osteomyelitis [13]. However, the impregnation of bone graft with AgNP has been poorly investigated, and microbiological studies with a focus on biofilm are lacking, including multidrug-resistant bacteria. The current literature about bone grafts and AgNP focuses on the development of implants and grafts using hydroxyapatites and other synthetic materials [14,15]. In addition to their antimicrobial properties, the potential of AgNPs to exhibit osteogenic activity makes this nanotechnology highly attractive, representing a significant innovation for bone grafting applications [16]. The design of scaffolds for bone regeneration demands materials that can both support rapid bone formation and provide adequate strength to withstand physiological loads without fracturing. Achieving a balance between mechanical integrity and bioactivity in a single material has proven challenging. However, advancements in nanotechnology, enabling the precise manipulation and characterization of materials at the nanoscale, hold the potential to revolutionize bone scaffold development, paving the way for solutions that outperform traditional autologous bone implants [17].

The use of bovine bone after processing and a cheap and easy method for AgNP impregnation has been poorly evaluated.

The purpose of this investigation was to evaluate the in vitro antibiofilm activity of a bovine bone impregnated with AgNP in the most important clinical pathogens, as well as the biocompatibility, cytotoxicity, and aspects of X-ray and CT-scan images.

## 2. Materials and Methods

### 2.1. Bone Processing

Spongy bone tissue discs were fabricated from adult bovine femur (metaphyseal portion), with dimensions of 6 mm in diameter and 3 mm in thickness, resulting in a volume of 84 mm³. This model was developed to facilitate the proposed analyses. Bovine bone tissue samples underwent a series of processing steps, including washing, agitation, sonication, centrifugation, and drying before impregnation with silver nanoparticles. These steps were carried out to remove various components such as bone marrow, blood cells, lipids, proteins, and nucleic acids. Each of these steps is detailed in a previous publication from our research group [18]. For processing, the volume of each washing solution was calculated at a ratio of eight samples to 10 mL of washing solution. Throughout the process, washes were performed using deionized water (Direct-Q 3 UV water purification system, Merck, Darmstadt, Germany). Hydrogen peroxide was used at a concentration of 30% (Synth, Diadema, Brazil), and the concentration of the sodium hydroxide used was 4% (Synth, Diadema, Brazil). The steps involved orbital agitation (shaker) at a constant temperature of 37 °C and 120 rpm (New Brunswick Innova^®^ 44, Merck, Darmstadt, Germany). Sonication was carried out at 35 °C with a frequency of 40 kHz (Sanders Medical, Santa Rita da Sapucaí, Brazil). Centrifugation at 2000× *g* was used to assist in removing debris, such as blood cells and residual washing agents. This processing method was adapted from NHS Blood and Transplant in the United Kingdom [19].

### 2.2. Silver Nanoparticles

**Figure 1:**
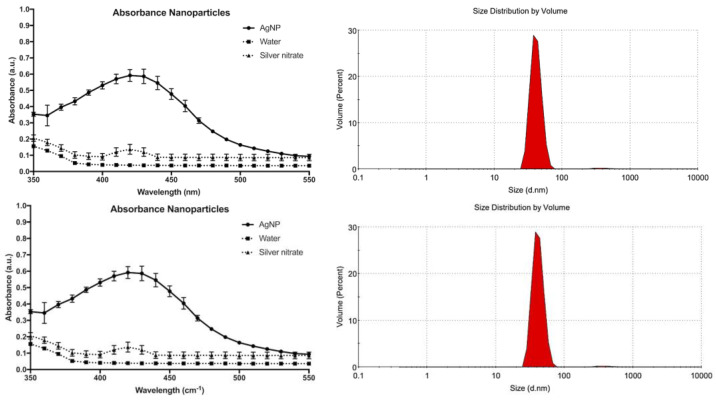
(**Left**) Absorbance spectrum of silver nanoparticles (AgNPs), water, and silver nitrate solution (control), showing a prominent plasmon resonance peak at approximately 450 nm, indicative of AgNP formation. The distinct peak at this wavelength corresponds to the characteristic surface plasmon resonance of AgNPs. (**Right**) Size distribution by volume of AgNPs obtained via dynamic light scattering (Malvern Zetasizer), revealing a predominant particle size of approximately 50 nm with a narrow distribution, demonstrating high uniformity in nanoparticle synthesis.

The procedure used to prepare 50 nm AgNPs was adapted from Dong et al. [20]. In the procedure, 8.5 mg of silver nitrate was dissolved (AgNO_3_) in 45 mL of deionized water, followed by the addition of 14.7 mg of trisodium citrate (Na_3_C_6_H_5_O_7_). Then, 5 mL of a glucose solution (200 mg in 10 mL of deionized water) was dripped into that solution and maintained under vigorous stirring at 60 °C. After one hour of stirring, the yellowish-colored solution contained the silver nanoparticles. Physical adsorption was used to incorporate the AgNPs into the bone matrix, based on an adaptation of the model reported by Becerril-Juárez et al. [21]. For this purpose, bone models were added to the AgNP solution at room temperature for 60 min, protected from light. After the incorporation of AgNPs into the bone matrices, the samples were kept at a temperature of 70 °C for 60 min in an oven with no air circulation system for drying, followed by lyophilization. In Figure 1, one can see the absorbance curve of the AgNP solution measured using a Versamax spectrophotometer (Molecular Devices, Sunnyvale, CA, USA). Nanoparticle morphology and surface charge were characterized using a Malvern Zetasizer. The size of the AgNPs was found to be 51.0 ± 2.2 nm, as measured by DLS (Dynamic Light Scattering) using Malvern Zetasizer (Figure 1 right). The surface charge on the particles was found to be –22.7 ± 5.5 mV, as measured by a Malvern Zetasizer.

### 2.3. Scanning Electronic Microscopy (SEM) and Energy-Dispersive X-Ray Spectroscopy (EDS)

SEM was used for the analysis of the morphology and microstructural characteristics before and after the impregnation of AgNPs in the bone matrix. The models were transferred to sterile glass Petri dishes with the primary fixing agent (0.68 g of 99.5% sucrose, 0.42 g of 98% sodium cacodylate, 0.6 mL of 30% glutaraldehyde (Merck, Darmstadt, Germany) in 19.4 mL of deionized water and left in contact for 45 min. After exposure to this primary fixing agent, the models were transferred every 10 min to the following solutions: buffer (composed of sucrose and sodium cacodylate at the concentrations mentioned above), 35% ethanol, 50% ethanol, 70% ethanol, 100% ethanol, and PA HMDS (hexamethyldisilazane) (Merck, Darmstadt, Germany). Following fixation, the models were kept in a desiccator until they were ready for observation with the SEM. They were pre-coated with gold particles in metal coating equipment with a Q150R ES^®^ rotary pump (Quorum Technologies, Lewes, UK) and subsequently mounted on a metal base for observation in a JEOL JSM 6010PLUS-LA scanning electron microscope at an acceleration voltage of 20 kV. Observations were performed at magnifications ranging from 2000 to 100,000 times, with AgNPs becoming visible at a magnification of 100,000×.

EDS is an analysis performed by an instrument coupled to the SEM, which allows for the measurement of the chemical elements present in the sample, as well as the determination of their concentrations with great precision. The bone discs were characterized by EDS to verify the presence and distribution of silver on the bone tissue. The EDS analysis was conducted at the JEOL JSM 6010PLUS-LA Analysis Station with an acceleration voltage of 20 keV.

### 2.4. Silver Nanoparticle Minimum Inhibitory Concentration (MIC)

A silver nanoparticle solution in progressive titers from 0.25 to 512 mg/L was used [22]. In total, 200 µL aliquots of silver nanoparticle solution in Mueller–Hinton broth in different concentrations were placed in a 96-well plate, and 5 µL of the solution of each microorganism (*Staphylococcus aureus* (ATCC^®^25923™), *Pseudomonas aeruginosa* (ATCC^®^25923™), *Candida albicans* (ATCC^®^10231™), *Enterococcus faecalis* (ATCC^®^29212™), multi-resistant *Acinetobacter baumannii* OXA-23 (clinical isolate), and *Escherichia coli* (ATCC^®^25922™)) was inoculated to reach a concentration of 10^6^ colony-forming units (CFUs)/mL. After incubation for 24 h at 36 °C, the MIC was defined as the lowest concentration that did not show bacterial growth. All tests were performed in triplicate [22].

### 2.5. Silver Nanoparticle Minimal Bactericidal Concentration (MBC) and Minimal Fungicidal Concentration (MFC)

MBC and MFC for AgNP were performed with the same microorganisms above, plating in triplicate 100 uL of all the solutions over the MIC. The MBC and MFC were determined as the final concentrations without bacterial or fungal growth.

### 2.6. Disc Diffusion Tests for AgNP Susceptibility

Two bone models, one without impregnation (negative control) and one with AgNPs (test), were transferred to Mueller–Hinton agar plates inoculated with the different bacteria and C. albicans for 24 h at 36 °C to check for the presence of an inhibition halo. The analysis was quantitative by measuring the diameter of the halo formed on the agar plate. For this analysis, the process was adapted from the experiment by Saegeman et al. (2008), such that the microorganisms described in the previous section were seeded on Mueller–Hinton agar plates (BD, Heidelberg, Germany), previously diluted to a turbidity standard equivalent to 0.5 McFarland [23].

### 2.7. Quantification of Biofilm Production on Plate

A suspension of 1.5 × 10^6^ CFU/mL of each microorganism was prepared using TSA (trypticase soy agar). Then, 200 uL was discharged into each well of a 96-well microplate (flat bottom). The microplate was incubated for 24 h at 36 °C under shaking conditions (120 rpm). After that, the biofilm of each tested strain was subjected to quantification by violet crystal (biomass) using a Versa-Max microplate ELISA reader (Molecular Devices, Sunnyvale, CA) and a wavelength of 570 nm [24].

### 2.8. Biofilm Production on Bone

From each microbe suspension, a 1:10 dilution was prepared in TSB broth until concentrations of 10^7^ CFU/mL of bacteria and 10^5^ CFU/mL of *C. albicans* were achieved [25]. Then, 10 mL of TSB broth was poured into sterile 12-well plates until it covered completely the bone discs (control and AgNP-impregnated) for 2 h under agitation (120 rpm). The specimens were transferred to a new sterile 12-well plate containing 0.9% NaCl to remove planktonic cells from the material. Then, specimens were transferred to another sterile 12-well plate and submerged in 10 mL of TSB broth at 37 °C for 24 h without agitation. During this step, the cells adhered to the device surface, forming the biofilm. After this step, the specimens were submerged into 50 mL conical tubes filled with 10 mL of sterile 0.9% NaCl to remove the residues and unadhered/planktonic cells (Step I). After this washing step, the specimens were allocated into 50 mL conical tubes filled with 10 mL of 0.9% NaCl for further processing (sonication), and the liquid of the last washing was stored for planktonic cell analysis (Step II).

### 2.9. Quantitative Analysis of Sessile Cells from the Biofilm

Five specimens of each group were transferred to sterile conical tubes with 10 mL of 0.9% NaCl and sonicated for 5 min in an ultrasonic bath using a Soniclean 15 (Sanders Medical, Santa Rita do Sapucaí, Brazil) at a frequency of approximately 40 kHz and temperature of 35 °C [26]. After the sonication step (step II), the supernatant (100 μL) was inoculated in TSA agar for growth evaluation and cell counting (in CFU/mL).

### 2.10. Cone Beam Computer Tomography (CBCT) and Digital Radiograph

For CBCT scans and digital radiographs, the bone scaffold was placed in a prepared and empty mandibular socket of the human dry mandible. This human dry mandible model was used previously [27]. Moreover, for CBCT scans and digital radiographs, the mandible was fixed at the base of a plastic container filled with water to simulate soft tissue (Influence of exposure parameters on the detection of simulated root fractures in the presence of various intracanal materials [28]). 

### 2.11. Direct Contact Cytotoxicity Assay

The cytotoxicity of treated cancellous bovine bone tissue was tested according to ISO 10993/5 [29]. The 3T3 mouse cell line (ECACC—European Collection of Authenticated Cell Cultures, clone A31) was seeded in 24-well plates at a density of 4.4 × 10^4^ cells/well. A total of 500 µL DMEM (Dulbecco’s Modified Eagle Medium) (Gibco Invitrogen^®^, Waltham, MA USA) + 2 mM glutamine + 10% fetal bovine serum were dispensed into each well. The plates were incubated for 48 h at 37 °C in a humidified atmosphere with 5 ± 1% CO_2_. Growth media were removed from the wells and replaced with 1.8% agar with 0.01% neutral red dye. DMEM and SDS (200 µg/mL) were used as negative and positive controls, respectively. After incubation, the cells in contact with the tissue were viewed under a phase contrast microscope (Nikon Eclipse TS 100; Nikon, Tokyo, Japan) for qualitative morphological analysis.

### 2.12. Biocompatibility in Animal Model

This study was approved by the Ethics Committee, and all procedures followed the regulations described in the national animal protection law. Ten male C57BL/6N mice (FIOCRUZ, Curitiba, PR, Brazil) were included in this study and distributed into two study groups: AgNP bovine graft (*n* = 4), negative control (autologous bone) (*n* = 4). The sample size was calculated as previously described and was also based on the recommendation of the ethics committee [30]. The mice were anesthetized, skin incisions were made on the top of their heads, and the periosteum was detached. Then, 5 mm diameter bone defects were created using a low-speed dental motor and a 5 mm diameter trephine drill (Implatex, Tokyo, Japan) (ABE 2018). All bone tissue was crushed with a bone crusher, and fragments of 1 mm were used after being passed through the pharmacological pattern sieve. In total, 50 mg of bone was implanted at the defect site. After the implants, the skin was sutured. The mice were monitored for 8 weeks before scheduled euthanasia. After euthanasia, the skull and surrounding soft tissues were immersed in 10% formalin for subsequent histological analysis with hematoxylin-eosin stain.

### 2.13. Graft Resorption by Computed Tomography (CT-Scan) in Animal Model

For the evaluation of osteointegration, high-resolution tomography was performed (CS 8200 3D, Atlanta, GA, USA). The images obtained were subjected to 3D reconstruction using the 3D Slicer system (Version 5.6.2), a free, open-source software program (BSD-style license). After reconstruction, the defects were measured through Autodesk Fusion 360, and the area was measured for comparison. The animals included in this study had 8 weeks of graft implant.

### 2.14. Statistical Analysis

For comparing the cell count in the groups, the median CFU/mL obtained with quantitative culture was analyzed by the Mann–Whitney test and presented with a median with interquartile range (25–75%). The difference in CFU/mL was significant when *p* < 0.05. The resorption area by computed tomography was compared in mm^2^, and the Mann–Whitney test was performed with a 25–75% interquartile range. The data were calculated, analyzed, and plotted using Prism 7.0 (Graphpad, San Diego, CA, USA).

## 3. Results

### 3.1. AgNP Synthesis, EDS, and SEM

The procedure used to obtain AgNPs was based on a green synthesis method that uses glucose, a nontoxic reducing agent, to convert Ag^+^ ions to Ag at zero oxidation state (Ag0). The final suspension presented a maximum absorption peak of around 420 nm (Figure 1), due to the plasmon band, confirming the formation of AgNPs of about 50 nm in diameter.

**Figure 2:**
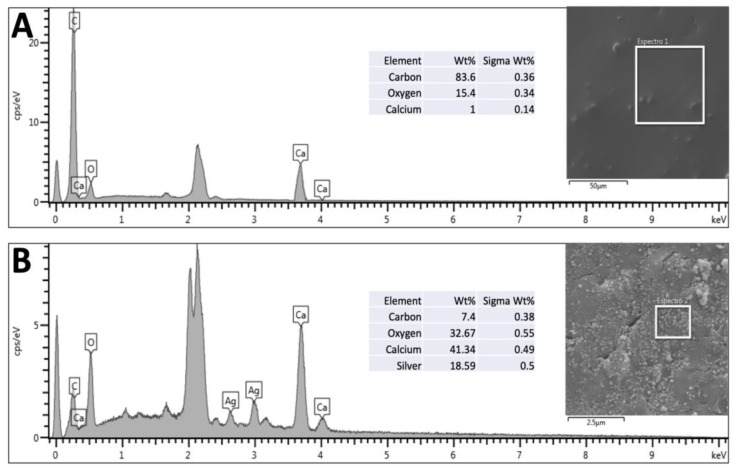
An energy-dispersive X-ray spectroscopy (EDS) analysis of bone samples. (**A**) The EDS spectrum of the unmodified bone, showing peaks corresponding to the primary elements present: carbon (83.6%), oxygen (15.4%), and calcium (1%), consistent with the organic and inorganic composition of bone. The inset image highlights the area analyzed (scale bar: 50 µm). (**B**) The EDS spectrum of bone impregnated with silver nanoparticles (AgNPs). Additional peaks for silver (18.59%) are evident, confirming the successful incorporation of AgNPs into the bone matrix. The elemental composition also reveals an increase in oxygen (32.67%) and calcium (41.34%), with reduced carbon content (7.4%) compared to the untreated bone. The inset image shows the corresponding analyzed region with higher magnification (scale bar: 2.5 µm).

**Figure 3:**
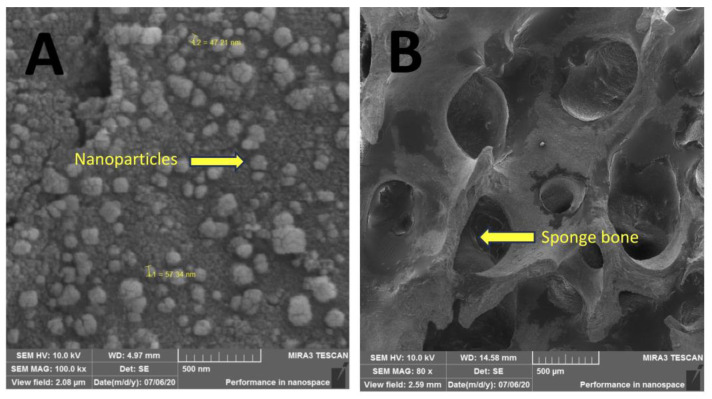

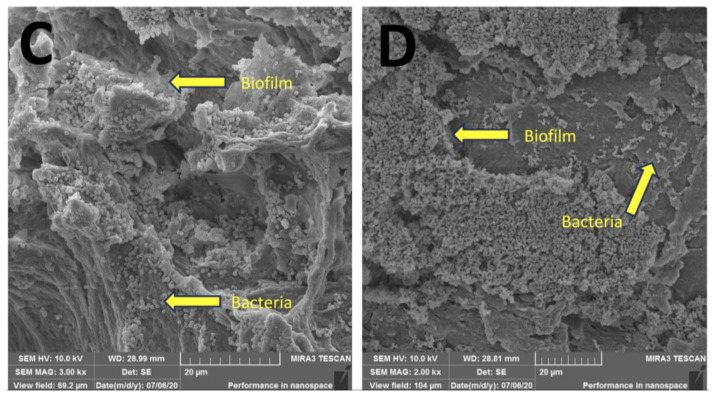
The Scanning Electron Microscopy (SEM) images of silver nanoparticle (AgNP)-impregnated bone and control samples. (**A**) A high-magnification (×100,000) view of the AgNPs impregnated on the bone surface, showing nanoparticle sizes ranging between 47 nm and 57 nm. (**B**) An SEM image of the processed bone sample before AgNP impregnation and biofilm formation, showing the porous sponge-like structure of the bone (×80). (**C**) A low-magnification view (×22) of the biofilm and bacterial colonization on the processed bone sample prior to AgNP impregnation. (**D**) An SEM image (×3000) of biofilm and bacterial colonization on the AgNP-impregnated bone sample, showing reduced microbial density compared to the control.

Using SEM and EDS, it is possible to observe the presence of nanoparticles impregnated in the bone scaffolds (Figure 2) due to the presence of two characteristic peaks of silver atoms, corresponding to 18.59% (*w*/*w*) of the sample. From SEM, one can confirm that the physical adsorption procedure used here was efficient in impregnating AgNPs and keeping them attached after drying the scaffolds (Figure 3). The 80× magnification represents the bone tissue after processing, showing that all peribone tissue was removed, and in Figure 3B is represented the bone sample used for microbiological tests (Figure 3C). In Figure 3A, the 100,000× magnification demonstrates the presence of AgNPs in the bone tissue, allowing the measuring of the particle size, which ranged from 47 to 57 nm in several measures. However, other sizes were found in the scanning, suggesting that the AgNP synthesis was not uniform. In Figure 3D is detailed the biofilm formation in the AgNP and control groups (non-impregnated bone) for the microbiological studies.

### 3.2. Silver Nanoparticles MIC and MBC

All pathogens were susceptible to AgNPs with low MIC (0.25–4 mg/L). The MIC of *S. aureus* and *P. aeruginosa* were 4 mg/L; 2 mg/L to *E. coli*, *E. faecalis*, and *A. baumannii*; and 0.25 to *C. albicans*. The susceptibility to silver allows the correct interpretation of the AgNP activity on the biofilm, avoiding false-negative results.

The MBC was the same as the MIC for *E. coli*, *S. aureus*, and *A. baumannii* (4 mg/L, 4 mg/L, and 8 mg/L, respectively). For *P. aeruginosa*, the MBC was twice the MIC (8 mg/L) and four times for *E. faecalis* (16 mg/L). For *C. albicans*, AgNPs did not achieve a fungicidal concentration, only fungistatic (>128 mg/L).

### 3.3. Quantification of Biofilm Production on Plate

**Figure 4:**
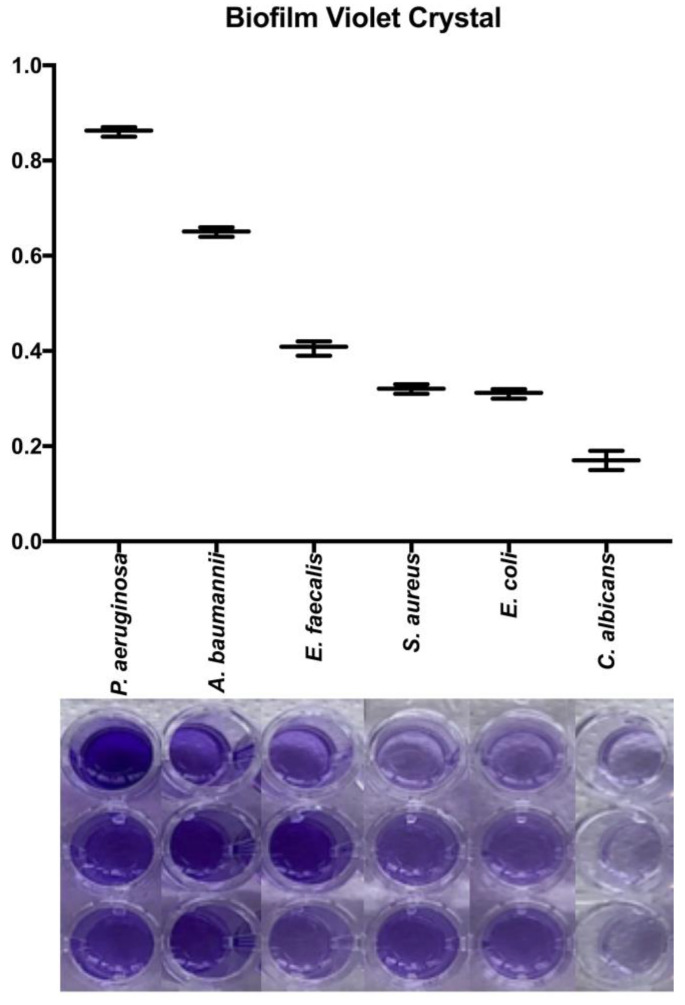
The quantification of biofilm on a plate of different microorganisms used in the AgNP-impregnated bone model. The measure of the absorbance biomass for each microorganism after a violet crystal assay. The biomass of each microorganism used in the biofilm model in the 96-well plate, before the measure of the absorbance (left axis).

All microorganisms produced biofilm in the plate test. The biomass of each microorganism varied significantly. Pseudomonas aeruginosa and A. baumannii were the most biofilm-producing microorganisms. In Figure 4 is detailed the macroscopic aspect of the biomass using a violet crystal assay (B) and the absorbance results using a microplate ELISA reader. The biomass evaluation by crystal violet showed a higher biofilm production in P. aeruginosa, implants with an absorbance of 0.86 ± 0.01, followed by *A. baumannii* (0.61 ± 0.01), *E. faecalis* (0.40 ± 0.01), *S. aureus* (0.32 ± 0.01), *E. coli* (0.31 ± 0.01), and *C. albicans* (0.17 ± 0.01). All microorganisms showed differences between them in biofilm production (*p* < 0.05), except for *S. aureus* and *E. coli* (*p* = 0.93).

### 3.4. The Quantitative Analysis of Sessile Cells from the Biofilm

**Figure 5:**
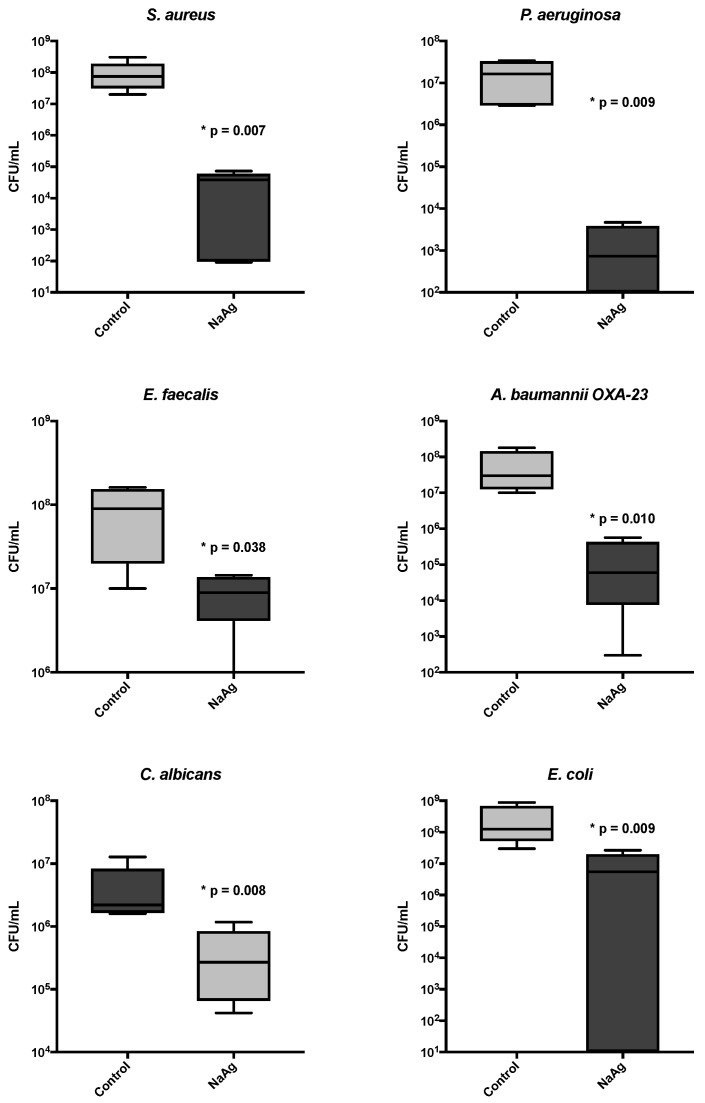
The cell count (CFU/mL) of biofilm in bone with AgNP and without impregnation (control). Boxplots comparing the colony-forming units (CFUs/mL) of various microorganisms in the control and silver nanoparticle (AgNP)-treated samples. *Staphylococcus aureus* shows a significant reduction in CFU/mL with AgNP treatment (*p* = 0.007). *Pseudomonas aeruginosa* exhibits a significant decrease in CFU/mL in the AgNP-treated group (*p* = 0.009). *Enterococcus faecalis* demonstrates reduced CFU/mL after the AgNP treatment (*p* = 0.038). *Acinetobacter baumannii* OXA-23 shows a significant suppression of CFU/mL with the AgNP treatment (*p* = 0.010). *Candida albicans* reveals a significant decrease in CFU/mL following the AgNP treatment (*p* = 0.008). *Escherichia coli* exhibits a marked reduction in CFU/mL in the AgNP-treated group (*p* = 0.009). The logarithmic scale is used to display CFU/mL for clarity, highlighting the antimicrobial effect of AgNPs compared to the control group.

The scaffold impregnated with AgNP presented a significant reduction in the biofilm cells for all microorganisms, reducing by more than 3 logs in CFU count. We evaluated the correlation of silver MIC with biomass and sessile cell count; however, no correlation was found (Figure 5 and Table 1).

### 3.5. CBCT and Digital Radiograph

**Figure 6:**
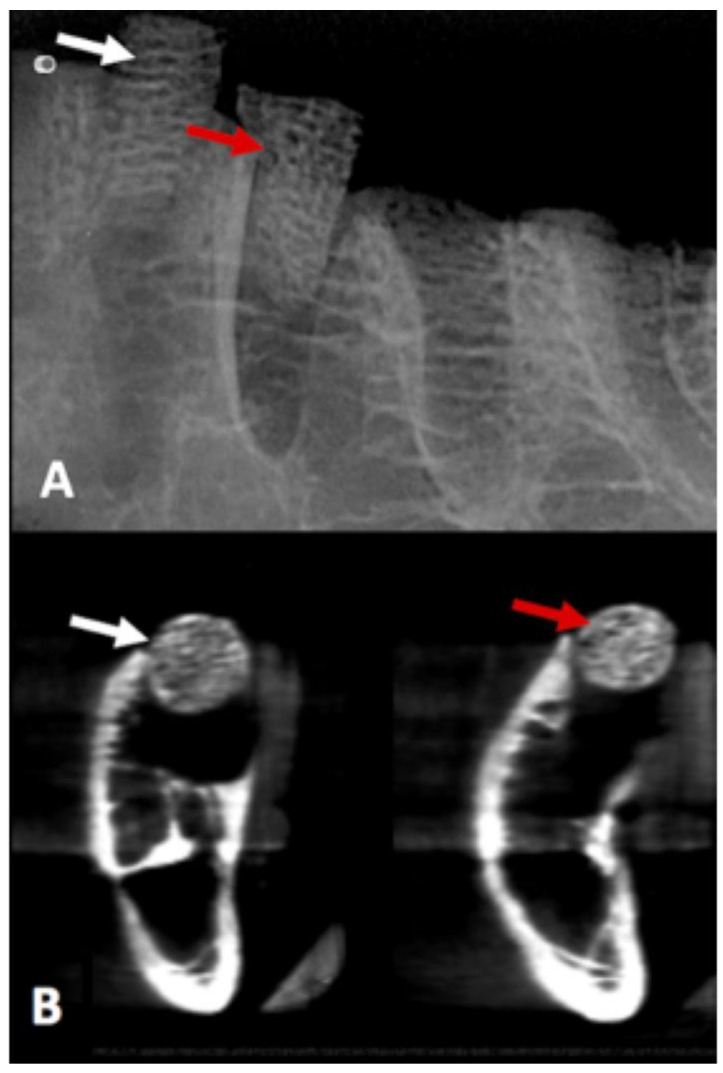
The white arrow shows the bone disc without AgNP impregnation, and the red arrow shows the bone disc with AgNP impregnation at the periapical digital radiograph (**A**) and CBCT scan (**B**). The radiographs demonstrated no difference in radio-opacity between the graft impregnated or not with AgNP.

Values obtained to compare the probability distributions of radiopacity bone disc, with and without AgNP impregnation, indicated that there were no different distributions of variables under the experimental conditions (*p* = 0.531) (Figure 6).

### 3.6. Direct Contact Cytotoxicity Assay

**Figure 7:**
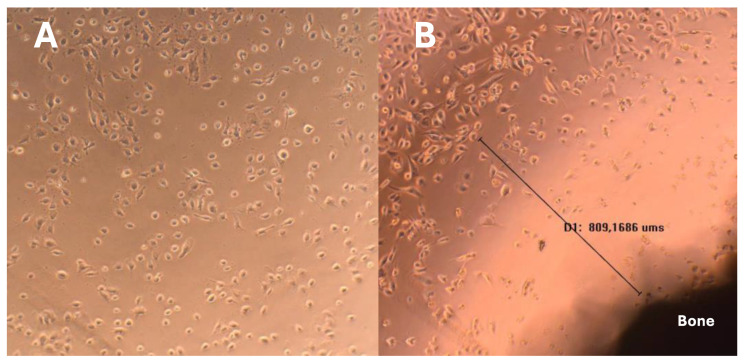
Cytotoxicity assay images of the bone scaffold with silver (magnification: (**A**,**B**) = 10×). (**A**) represents the negative control and (**B**) the cytotoxicity assay results of the treated bone. The black line represents the distance between the bone sample and the viable cell zone.

A direct contact cytotoxicity assay revealed that the cell monolayer next to the bone impregnated with AgNP was destroyed. The presence of a halo around the sample indicating cell death was verified through the analysis of images under 10× (Figure 7B). Furthermore, the presence of evident morphological changes and round cells during lysis was observed. The monolayer was not destroyed, indicating a moderate reactivity and degree of toxicity. A halo of a 922.6 ± 236.5 μm diameter indicated a moderate reactivity and degree of toxicity.

### 3.7. Biocompatibility in Animal Model

**Figure 8:**
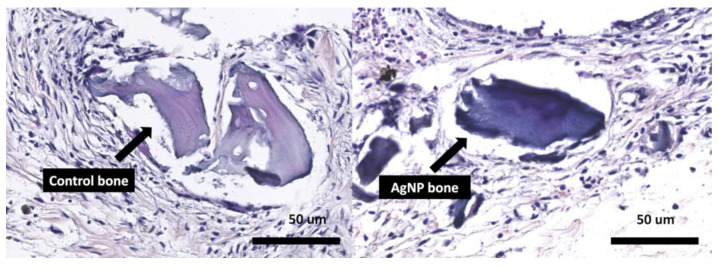
A histological comparison of the bone samples with and without silver nanoparticle (AgNP) impregnation. The biocompatibility analysis in a mouse calvaria model 8 weeks after implantation, comparing a control group with autologous bone (**left**) and bone with AgNPs (**right**). The grafts (arrows) are highlighted in the image, and it is possible to observe the formation of fibrosis around the graft in a similar manner between the two types of grafts, demonstrating the biocompatibility of the bone with AgNPs.

The animal study involved a comparison between a group with grafts containing AgNPs and another group using the animal’s own bone. Autologous bone grafting involves the removal of a portion of the skull cap, followed by its fragmentation and subsequent implantation. In Figure 8, it is possible to identify the bone graft, both the negative control and the AgNP-treated bone, surrounded by fibroblasts, with no acute inflammatory response, an absence of neutrophils, macrophages, and only a few mononuclear cells within a fibrotic area. In comparison with the control group, there was no difference in biocompatibility (Figure 8).

**Figure 9:**
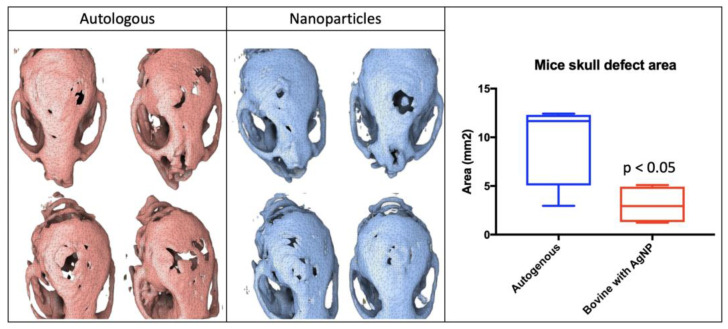
The evaluation of defect healing in mice skulls treated with autologous bone grafts or bovine bone grafts impregnated with silver nanoparticles (AgNPs). Left Panel (Autologous Bone): Three-dimensional reconstructions of skulls treated with autologous bone grafts, showing the incomplete healing of the defect areas. Middle Panel (Nanoparticles): Three-dimensional reconstructions of skulls treated with bovine bone grafts impregnated with AgNPs, demonstrating improved healing and smaller defect areas compared to the autologous group. Right Panel (Mice Skull Defect Area): A quantitative comparison of the defect areas in mm² between the two treatment groups. The AgNP-treated group exhibited significantly smaller defect areas (*p* < 0.05) compared to the autologous bone group. This figure highlights the enhanced bone regeneration potential of AgNP-impregnated bovine grafts in promoting healing in critical-sized skull defects in mice. Bone resorption analysis in a mouse calvaria model 8 weeks after implantation, comparing a control group with autologous bone and bone with AgNPs. The area was measured in mm^2^.

The resorption area in the autograph as per computed tomography was 11.7 mm^2^ and 2.9 mm^2^ in the AgNP graft (*p* < 0.05) (Figure 9).

## 4. Discussion

The development of a bone substitute with the potential for antimicrobial activity has always been important in orthopedics and dentistry. In orthopedics, it serves the purpose of filling spaces that may be associated with biofilms, while in dentistry, it addresses the potential risk of contamination in the field of implantology. The choice of silver as a potential antimicrobial agent was based on its broad spectrum of activity against both gram-negative and gram-positive bacteria; its ability to be transformed into nanoparticles to enhance its activity, a theoretical potential for improved osteointegration; and furthermore, its lack of chemical relation to antibiotics used in treatments, thereby avoiding the induction of resistance or the selection of multidrug-resistant bacteria through microbiota modification [31].

In our study, all pathogens were susceptible to AgNPs with low MICs. These values were similar to those found by other authors [32]. Due to potential variations in nanoparticle preparation during production, MIC values can be varied among the studies. Mathur et al. described the antibacterial action of AgNPs through penetration into the microorganism’s cell membrane, increasing its permeability, leading to the rupture of this structure and consequent cell death [33]. Another mechanism described is the formation of free radicals that damage the cell membrane, causing porosity and cell lysis [34].

In recent years, there has been a significant increase in studies using AgNPs with antibiofilm activity. These studies have assessed the antibiofilm activity of AgNPs in combination with metallic surfaces such as titanium compounds, aiming to prevent infections associated with orthopedic prostheses as well as dental implants [35]. In the context of dentistry, the combination of AgNPs with restorations has shown activity against *Streptococcus* biofilms, the primary pathogen associated with dental biofilms and subsequent caries formation [36]. Another antibiofilm strategy involving the use of AgNPs is their combination with other biocompatible materials like chitosan, enabling their integration into hydrogels or 3D printing using bioprinters, facilitating the development of therapeutic or preventive wound dressings [37,38].

Smaller particles have a higher surface area that allows the release of a higher amount of silver ions, responsible for the most acceptable mechanism of bactericidal action [39]. In this sense, smaller particles are expected to present a greater antimicrobial effect than bigger particles and particle aggregates. This could be an explanation for the maintenance of viable bacterial cells after scaffold impregnation, mainly *E. faecalis* and *E. coli*. Bacteria with a high capacity for multiplication and biofilm production, like *P. aeruginosa*, *S. aureus*, and *A. baumannii* showed higher susceptibility to AgNPs. This can be highly beneficial if our AgNP-treated graft is used as an adjunct in chronic osteomyelitis cases requiring filling [40].

Olson et al. highlighted that *P. aeruginosa*, *E. coli*, and *S. aureus* produce biofilms in a short period of time and without great nutritional requirements, data similar to those obtained in our study [41]. However, in our study, the broth used in the biofilm model presents abundant nutrients, which can differ in the biofilm pattern and bacterial redox system, which can affect silver activity [42]. Anuj et al. evaluated the association of silver nanoparticles with linezolid as a bactericidal agent on *E. coli* MTCC^®^443™, where the resistance mechanism of the drug efflux of this microorganism was inactivated by AgNP [43]. This is an important issue, considering that AgNPs can be an important molecule to be used in combination with antibiotics. Grafts with antimicrobial action available typically consist of biotic molecules, such as bioactive glass. However, the cost of these materials is extremely high, and we believe that the graft developed here may offer similar benefits [44].

Toxicity does not pertain to bacterial cells only but also to eukaryotic cells, as nanoparticles may exhibit some degree of cytotoxicity [45,46]. The cytotoxicity assay showed moderate toxicity in the AgNP-impregnated bone scaffold we developed, similar to previous cited publications. Another issue with the use of silver is its discoloration, which can stain teeth or skin when applied directly to these tissues [47]. However, in our study, it is used as an implant, which would not cause aesthetic damage.

Despite the moderate cytotoxicity, in the biocompatibility test using a mouse calvaria model, it was evident that there was no difference in the inflammatory response or bone resorption after 8 weeks of evaluation. The biocompatibility of silver-containing nanoparticles has already been demonstrated, which reinforces the possibility of using this material in medical practice [48]. Silver nanoparticles, despite being cytotoxic, can stimulate the development of osteoblasts and be associated with increased osteogenesis [49]. This is the reason why the graft with nanoparticles showed less resorption compared to the autologous bone group in the CT reconstruction.

This study had some limitations because we evaluated a few bacteria, which could include multidrug-resistant isolates. Biomechanical evaluation can be important for a structural scaffold if used for large bone defects. The shelf life was not determined beyond six months. Thus, as a product, a more extended time (12 months) would be important. The cone beam tomography showed no difference in the distributions of variables under the experimental conditions, suggesting that AgNP does not interfere in radiological exams, an important characteristic for scaffolds in dental implants. Even though the literature is broad on impregnated materials, microbiological studies with biofilm on bovine scaffolds have not been reported yet.

## 5. Conclusions

In conclusion, this study demonstrated the successful synthesis and incorporation of AgNPs into bone scaffolds using a green synthesis method with glucose. The AgNP-impregnated scaffolds showed promising antimicrobial properties, significantly reducing biofilm production and sessile cell counts across various tested microorganisms while maintaining biocompatibility in both in vitro and in vivo models. Despite moderate cytotoxicity observed in direct contact assays, the AgNP-treated scaffolds exhibited enhanced bone regeneration in an animal model, as evidenced by the improved healing and reduced defect areas compared to autologous bone grafts. These findings highlight the potential of AgNPs as a valuable addition to bone scaffolds for applications in tissue engineering and infection control.

## Figures and Tables

**Table 1:** Correlations of biofilm on plate, MIC (minimal inhibitory concentration in mg/L), and biofilm on bone (control and with silver nanoparticles [AgNP]) with respective Log Reduction. Absorbance of biofilm on plate was used as comparative for *p* value.

	Biofilm on Plate		MIC		Bone Biofilm	
Microorganism	Absorbance (ƛ)	ƛ/Negative Control		Control	AgNP	Log Reduction
*P. aeruginosa*	0.86	15.7	4	1.6 × 10^7^	7.3 × 10^2^	4.35
*E. coli*	0.31	5.7	2	1.3 × 10^8^	5.4 × 10^6^	1.36
*S. aureus*	0.32	5.8	4	7.5 × 10^7^	3.7 × 10^4^	3.30
*E. faecalis*	0.41	7.4	2	9.0 × 10^7^	9.1 × 10^6^	1.00
*A. baumannii* OXA-23	0.65	11.8	2	3.0 × 10^7^	6.0 × 10^4^	2.70
*C. albicans*	0.17	3.1	0.25	2.2 × 10^6^	2.7 × 10^5^	0.91
Negative Control	0.06	1.0	-	-	-	-
*p* value			0.240	0.983	0.629	0.078

## Data Availability

Data available on request from the authors.
